# A primary *Chlamydia trachomatis* genital infection of rhesus macaques identifies new immunodominant B-cell antigens

**DOI:** 10.1371/journal.pone.0250317

**Published:** 2021-04-22

**Authors:** Arlo Randall, Andy Teng, Xiaowu Liang, Sukumar Pal, Alice F. Tarantal, Joseph Fike, Peter A. Barry, Luis M. de la Maza

**Affiliations:** 1 ImmPORT Therapeutics, Inc./Antigen Discovery Inc., Irvine, California, United States of America; 2 Department of Pathology and Laboratory Medicine Medical Sciences I, University of California, Irvine, California, United States of America; 3 California National Primate Research Center, University of California, Davis, Davis, California, United States of America; 4 Pediatrics, Cell Biology and Human Anatomy, School of Medicine, University of California, Davis, Davis, California, United States of America; 5 Center for Comparative Medicine, University of California, Davis, Davis, California, United States of America; 6 Departments of Pathology and Laboratory Medicine, University of California, Davis, Davis, California, United States of America; New York State Department of Health, UNITED STATES

## Abstract

To identify immunodominant antigens that elicit a humoral immune response following a primary and a secondary genital infection, rhesus monkeys were inoculated cervically with *Chlamydia trachomatis* serovar D. Serum samples were collected and probed with a protein microarray expressing 864/894 (96.4%) of the open reading frames of the *C*. *trachomatis* serovar D genome. The antibody response to the primary infection was analyzed in 72 serum samples from 12 inoculated monkeys. The following criteria were utilized to identify immunodominant antigens: proteins found to be recognized by at least 75% (9/12) of the infected monkeys with at least 15% elevations in signal intensity from week 0 to week 8 post infection. All infected monkeys developed *Chlamydia* specific serum antibodies. Eight proteins satisfied the selection criteria for immunodominant antigens: CT242 (OmpH-like protein), CT541 (mip), CT681 (ompA), CT381 (artJ), CT443 (omcB), CT119 (incA), CT486 (fliY), and CT110 (groEL). Of these, three antigens, CT119, CT486 and CT381, were not previously identified as immunodominant antigens using non-human primate sera. Following the secondary infection, the antibody responses to the eight immunodominant antigens were analyzed and found to be quite different in intensity and duration to the primary infection. In conclusion, these eight immunodominant antigens can now be tested for their ability to identify individuals with a primary *C*. *trachomatis* genital infection and to design vaccine strategies to protect against a primary infection with this pathogen.

## Introduction

*Chlamydia trachomatis* is the most common notifiable disease in the USA and is thought to be the most common bacterial sexually transmitted infection (STI) worldwide [1, 2]. The patient population most affected are young sexually active individuals [2–6]. Primary, repeated and chronic infections can lead to long-term sequelae including pelvic inflammatory disease (PID), infertility and ectopic pregnancy [6–12]. Infected mothers can transfer *C*. *trachomatis* to their newborns that can develop ocular, respiratory and gastrointestinal infections [13–15]. In countries with poor hygienic conditions, particularly those located in Sub-Saharan Africa, *C*. *trachomatis* produces chronic ocular infections that can lead to trachoma, the most common preventable form of blindness [8, 11]. Decades ago, several countries implemented screening programs focusing mainly on young high-risk individuals. Surprisingly, increases in prevalence of genital infections have been reported in spite of antibiotic treatment [16, 17]. It has been postulated that the use of antibiotics may have interfered with the development of natural immunity [16]. Thus, vaccination may be the best approach to prevent infections with *C*. *trachomatis* [18–24].

To identify antigens that elicit humoral and cell-mediated immune responses several investigators have tested sera and T-cells from patients at different stages of a *C*. *trachomatis* infection [25–30]. Others have used the mouse model to discover immunodominant antigens following a *Chlamydia muridarum* infection [31–34]. In pigtailed macaques, immunodominant antigens were detected following multiple cervical and/or fallopian tube infections with *C*. *trachomatis* serovars D and/or E [35].

The goals of this study were to identify in rhesus monkeys unique B-cell immunodominant antigens following a primary *C*. *trachomatis* vaginal infection and also to compare primary and secondary infections antibody responses. This type of study cannot be performed in humans since it is quite difficult to precisely define the time of first exposure to *C*. *trachomatis*. Thus, using a whole genome protein microarray, we screened sera from naïve rhesus macaques (*Macaca mulatta*) cervically infected for the first time with *C*. *trachomatis* serovar D. We discovered eight antigens that were recognized by at least 75% (9/12) of the infected animals and that potentially can be used to identify individuals with a primary *C*. *trachomatis* infection. Furthermore, we compared the antibody responses to the immunodominant antigens following the primary and secondary challenges. These immunodominant antigens can now also be tested for their ability to induce protective responses in relevant animal models before implementation in a human chlamydial vaccine.

## Materials and methods

### Animals

Fifteen healthy sexually mature adult female rhesus monkeys (*Macaca mulatta*) were maintained at the California National Primate Research Center (CNPRC) at the University of California (UC), Davis. The animal care and use protocol for this study was approved prior to implementation by the Institutional Animal Care and Use Committee (IACUC). All procedures conformed to the requirements of the Animal Welfare Act. This study was carried out in strict accordance with the recommendations in the Guide for the Care and Use of Laboratory Animals of the National Institutes of Health and in accordance with the recommendations of the Weatherall report, ‘‘*The use of nonhuman primates in research*”. Activities related to animal care including housing, feeding, and environmental enrichment were performed in accordance with IACUC-approved standard operating procedures (SOPs) at the California National Primate Research Center (http://www.cnprc.ucdavis.edu). Monkeys were housed individually in stainless steel cages (6.4 square cage measuring 34 x 27 x 32) with a 4:00 am to 6:00 pm light cycle at a temperature of 25–27°C. Purina Monkey Chow and water were provided at libitum. Water was provided to each cage by rigid PVC pipes and a “lixit” device. Seasonal produce including cereal and seeds were provided as supplements. All food items were fresh. Multiple sources of enrichment were given including manipulation, food, visual and auditory activities. Cage enrichment devices were not made of bisphenol A plastic. All animals were checked twice a day to make sure they were healthy. Any changes in eating, drinking and physical activity were evaluated. Euthanasia was consistent with the recommendations of the American Veterinary Medical Association (AVMA) Guidelines on Euthanasia and Primate Center SOPs (overdose of pentobarbital). Animals (N = 15) were sedated with intramuscular (IM) ketamine (10–30 mg/kg) or telazol (5–8 mg/kg) for all procedures. To rule out previous or current chlamydial infection, all animals were prescreened by cervical culture and serology.

### *C*. *trachomatis* stocks

The human *C*. *trachomatis* serovar D (UW-3/Cx) was obtained from the American Type Culture Collection (ATCC; Manassas, VA) and was grown in HeLa-229 cells as described [36]. Density gradient-purified elementary bodies (EB) were stored at -80°C in 0.2 M sucrose, 20 mM sodium phosphate (pH 7.4), and 5 mM glutamic acid (SPG) [37].

### Infection of rhesus macaques with *C*. *trachomatis*

To synchronize the menstrual cycle, one month before infection, all animals were treated intramuscularly with 30 mg of medroxyprogesterone acetate (MPA) [38, 39]. Vaginal infection was performed by gently placing the inoculum on the os cervix through a speculum. One group of six rhesus monkeys received 10^7^ inclusion forming units (IFU) and a second group of six animals received 10^5^ IFU of *C*. *trachomatis* serovar D. Three additional monkeys were administered SPG and served as mock-infection controls. Three monkeys from the group infected with 10^5^ IFU and three monkeys from the group infected with 10^7^ IFU underwent scheduled necropsies at 10 weeks post-infection. The remaining nine animals received a second injection of MPA (30 mg) and at 14 weeks from the initial infection 6 of 9 monkeys were inoculated vaginally with 10^5^ IFU of *C*. *trachomatis* serovar D. Vaginal cultures were collected weekly (limit of detection 7 *C*. *trachomatis* IFU/culture). Blood was collected the day before each inoculation and at regular intervals following post-inoculation until the study end-point.

### Inclusion fluorescent-antibody assay

To determine the total IgG antibody titers, the inclusion immunofluorescent assay (IFA) was performed as previously described [40]. Briefly, McCoy cells in shell vials were infected with *C*. *trachomatis* serovar D (UW-3/Cx) and the monolayers were fixed with methanol at 40 h post infection. Monkey sera were serially diluted two-fold with PBS, starting at a 1:50 dilution and added to the monolayers. Secondary FITC-labeled anti-human IgG was incubated and chlamydial inclusions were quantified under a fluorescent microscope. Pre-infection sera were used as negative controls.

### Production of *C*. *trachomatis* microarray

The *C*. *trachomatis* protein microarray used in this study is identical to the one used in our prior study [35]. In both projects the array chips were manufactured by Antigen Discovery, Inc. Briefly, (1) protein coding genes were cloned into plasmids, (2) the proteins were expressed from these clones, and (3) were printed onto slides without purification.

### Microarray probing and data collection

A total of 72 serum samples from 15 monkeys, representing pre and post inoculation time points, were tested for antibodies at Antigen Discovery, Inc. using the *C*. *trachomatis* serovar D microarray with the identical protocol as in our prior project [35]. Briefly, 1) serum samples were deposited on arrays and incubated with biotin-conjugated secondary antibody; 2) the secondary antibody was detected using streptavidin conjugated to a fluorescent molecule, and 3) the arrays were scanned to quantify the fluorescence.

### Bioinformatics analysis

#### Data processing and normalization

Triplicate signals for each antigen were corrected for background noise (QuantArray software; Perkin Elmer) and normalized utilizing the variance stabilization and calibration for microarray data (VSN) package implemented in R [35, 41, 42]. The VSN model was built using only the 192 no-DNA control spot intensities for each sample. The VSN normalized data uses these control spots for sample specific adjustments and also transforms the data using the base-2 log scale, so each unit change corresponds to a doubling.

#### Statistical analysis

The VSN normalized data was used to determine the statistical significance of the increases in signal intensity following *C*. *trachomatis* inoculation by t-test using the ‘stats’ package implemented in R.

Paired t-tests were used to compare pre- and post-inoculation samples from the same subjects (n = 15). For each protein, the null hypothesis was that the mean of the intensities of the pre-inoculation samples was greater than or equal to the mean of the intensities of the post-inoculation samples [35]. The alternative hypothesis was that the mean of the post-inoculation samples was greater.

Independent sample t-tests were used to compare the control group (n = 3) to the infected group (n = 12). For each protein, the null hypothesis was that the mean of the intensities of the control group samples was greater than or equal to the mean of the intensities of the infected group samples. The alternative hypothesis was that the mean of the infected group samples was greater.

In Tables 1 and 2 summary statistics are presented for individual antigens and also for the average of all 894 ORFs on the array. The identifier “Avg-894-ORFs” is used to denote the average of all spots in the calculation. In Tables 3 and 4 changes in signal between longitudinal samples are categorized as: increases, stable, or decreasing using 15% change in signal as a threshold. If a signal changes less than 15% in either direction it is categorized as stable. For these calculations the VSNdata is transformed back to the linear scale (2^x where x is the VSN normalized data). When changes in signal are presented in terms of percentage changes the linear scale data is also used.

**Table 1:** Immunodominant antigens.

Protein ID *C*. *trachomatis*	NCBI Annotation	Positive Infected Monkeys	Week 0 to 4 Infected Average Increase as %	Week 0 to 8 Infected Average Increase as %	P-Value: Week 0 to Week 8 Average Increase	Week 14 to 18 Infected Average Increase as % (Boost Effect)	Protein ID *C*. *muridarum*	Predicted function	Mouse: J. Proteomics 77:176–186, 2012	Mouse: Microbes Infect. 14: 659–665, 2012	Humans: J. Immunol 185:1670–1680, 2010	Macaques: J. Proteomics 108:99–109, 2014
CT242	OmpH	10	75%	187%	1E-03	44%	TC513	OmpH	No	No	No	Yes
CT541	MIP	9	84%	120%	6E-03	16%	TC828	MIP	Yes	Yes	Yes	Yes
CT681	OmpA	10	64%	99%	4E-04	29%	TC052	MOMP	Yes	No	Yes	Yes
CT381	ArtJ	9	35%	91%	8E-03	19%	TC660	ABC	Yes	Yes	Yes	No
CT443	OmcB	12	49%	80%	3E-05	59%	TC727	60 kDa crp	Yes	Yes	Yes	Yes
CT119	IncA	11	22%	68%	6E-03	23%	TC396	IncA	Yes	No	Yes	No
CT486	FliY	10	19%	48%	1E-02	-3%	TC773	ABC	Yes	No	No	No
CT110	GroEL_1	9	17%	16%	8E-02	58%	TC386	60 kDa hsp	Yes	No	Yes	Yes
Avg-894-ORFs			2%	1%	9E-01	1%						

Eight proteins were defined as immunodominant using the following criteria: At least 75% of the infected monkeys (9/12) show an increase in antibody response from week 0 to week 8 that is greater than the maximum increase observed among the 3 controls, and the average increase from week 0 to week 8 is greater than 15%. The proteins are sorted by the average intensity increase of the infected monkeys at week 8.

**Table 2:** Immunodominant antigens: Comparison of primary and secondary infections.

Protein ID C. trachomatis	NCBI Annotation	*Primary to secondary (day of infection)*	Week 0 to 14 Count With Increase of 15+%	Week 0 to 14 Infected Average Increase as %	P-Value: Week 0 to Week 14 Average Increase	*Primary to Secondary (4 weeks after infections)*	Week 4 to 18 Count With Increase of 15+%	Week 4 to 18 Infected Average Increase as %	P-Value: Week 4 to Week 18 Average Increase
CT242	OmpH		5	78.4%	5E-02		4	78.9%	2E-02
CT541	MIP		3	81.0%	1E-01		3	27.2%	2E-01
CT681	OmpA		3	48.0%	8E-02		4	27.0%	1E-01
CT381	ArtJ		2	41.4%	2E-01		3	33.3%	2E-01
CT443	OmcB		5	74.7%	1E-02		4	66.2%	3E-02
CT119	IncA		5	64.8%	1E-01		4	62.2%	5E-02
CT486	FliY		4	44.4%	1E-01		4	32.3%	1E-01
CT110	GroEL_1		5	31.8%	3E-02		3	50.5%	8E-02
Avg-894-ORFS			1.1	-3.2%	7E-01		1.1	0.0%	9E-01

Results are presented for the 6 monkeys that were given both primary and secondary infections. Average antibody levels are higher on the day of secondary infection than primary for all 8 immunodominant antigens (p < 0.05 for CT242, CT443, and CT110). Average antibody levels are also higher 4 weeks after secondary infection than 4 weeks after primary infection for all 8 immunodominant antigens (p < 0.05 for CT242, CT443, and CT119).

**Table 3:** Percentages of signals that increase or decrease between corresponding weeks of primary infection and secondary infection.

Primary vs. Secondary Infection					
At time of infection (week 0 vs 14)			4 Weeks After Infection (week 4 vs 18)		
	8 Selected	All 894 ORFs		8 Selected	All 894 ORFs
Increase	66.7%	18.4%	Increase	60.4%	17.7%
Stable	24.2%	62.8%	Stable	25.0%	67.2%
Decrease	9.1%	18.8%	Decrease	14.6%	15.1%

A threshold of 15% change in the linear signal is used to categorize each change in signal between samples as (1) **increase:** change is > +15%; (2) **stable:** change is less than 15% in either direction; or (3) **decrease:** change is less than -15%. The column *8 Selected* presents the percentages of signal changes in each category for the 8 immunodominant proteins (48 measurements total: 8 proteins x 6 subjects). The column *All 894 ORFS* presents the category percentages (5364 measurements total: 894 proteins x 6 subjects).

**Table 4:** Percentages of signals that increase or decrease in consecutive samples.

**Primary Infection**					
Week 0 to 4			Week 4 to 8		
	8 Selected	All 894 ORFs		8 Selected	All 894 ORFs
Increase	64.6%	12.2%	Increase	39.6%	7.6%
Stable	26.0%	71.1%	Stable	35.4%	77.5%
Decrease	10.4%	16.7%	Decrease	25.0%	14.9%
**Secondary Infection**					
Week 0 to 4 (from 2nd infection)			Week 4 to 9 (from 2nd infection)		
	8 Selected	All 894 ORFs		8 Selected	All 894 ORFs
Increase	60.4%	7.7%	Increase	2.1%	3.9%
Stable	35.4%	85.6%	Stable	27.1%	84.8%
Decrease	4.2%	6.7%	Decrease	70.8%	11.3%

A threshold of 15% change in the linear signal is used to categorize each change in signal between samples as (1) increase: change is greater than +15%; (2) stable: change is less than 15% in either direction; or (3) decrease: change is less than -15%. The column 8 Selected presents the percentages of signal changes in each category for the 8 immunodominant proteins (48 measurements total: 8 proteins x 6 subjects). The column All 894 ORFS presents the category percentages (5364 measurements total: 894 proteins x 6 subjects).

## Results

### Vaginal cultures

**Fig 1:**
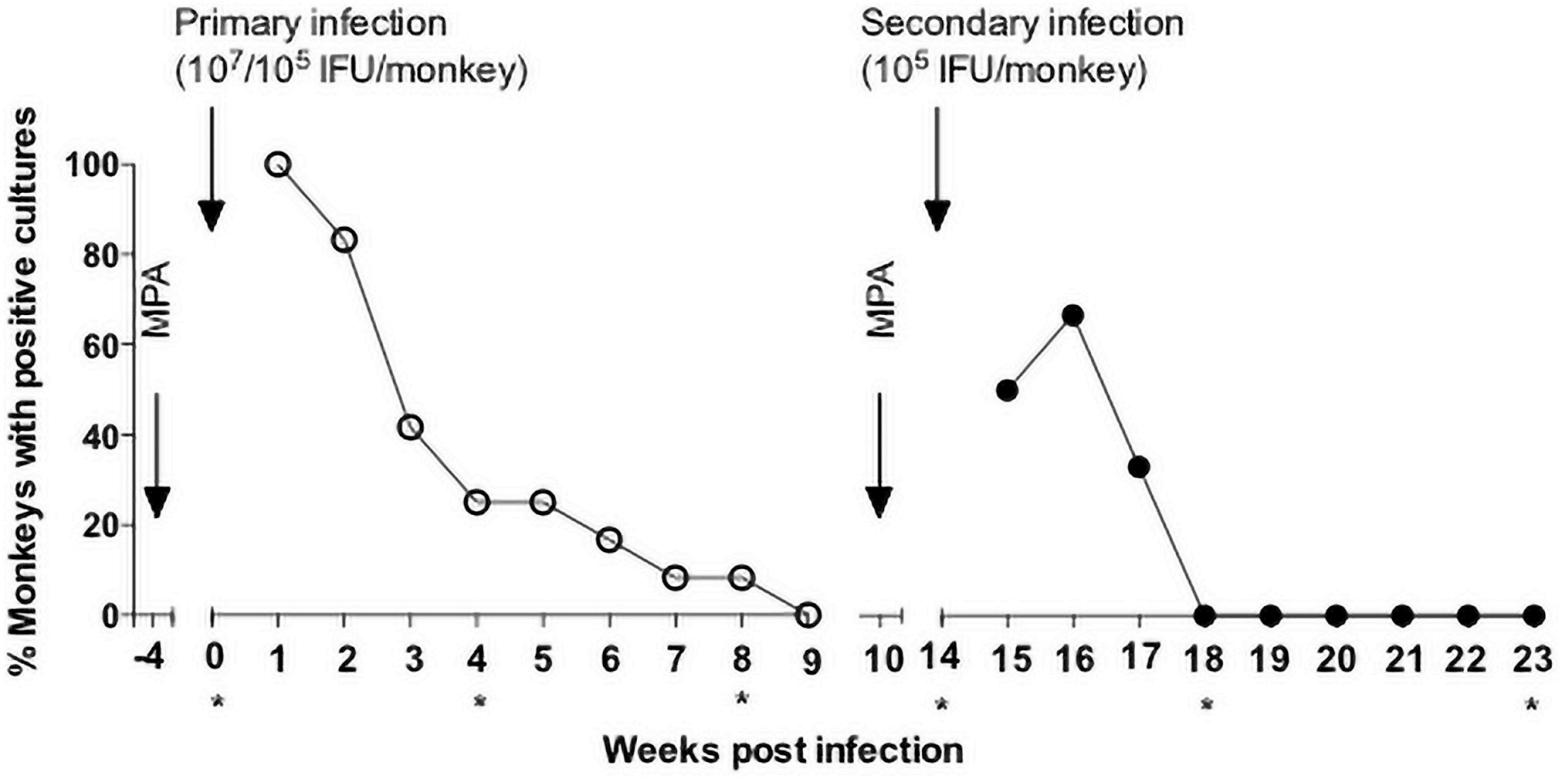
Vaginal cultures following primary and secondary *C*. *trachomatis* vaginal infections. Results of vaginal cultures collected from Rhesus monkeys following a primary and a secondary vaginal infection with *C*. *trachomatis* serovar D. *Times when sera was collected for testing with the microarray.

**Fig 2:**
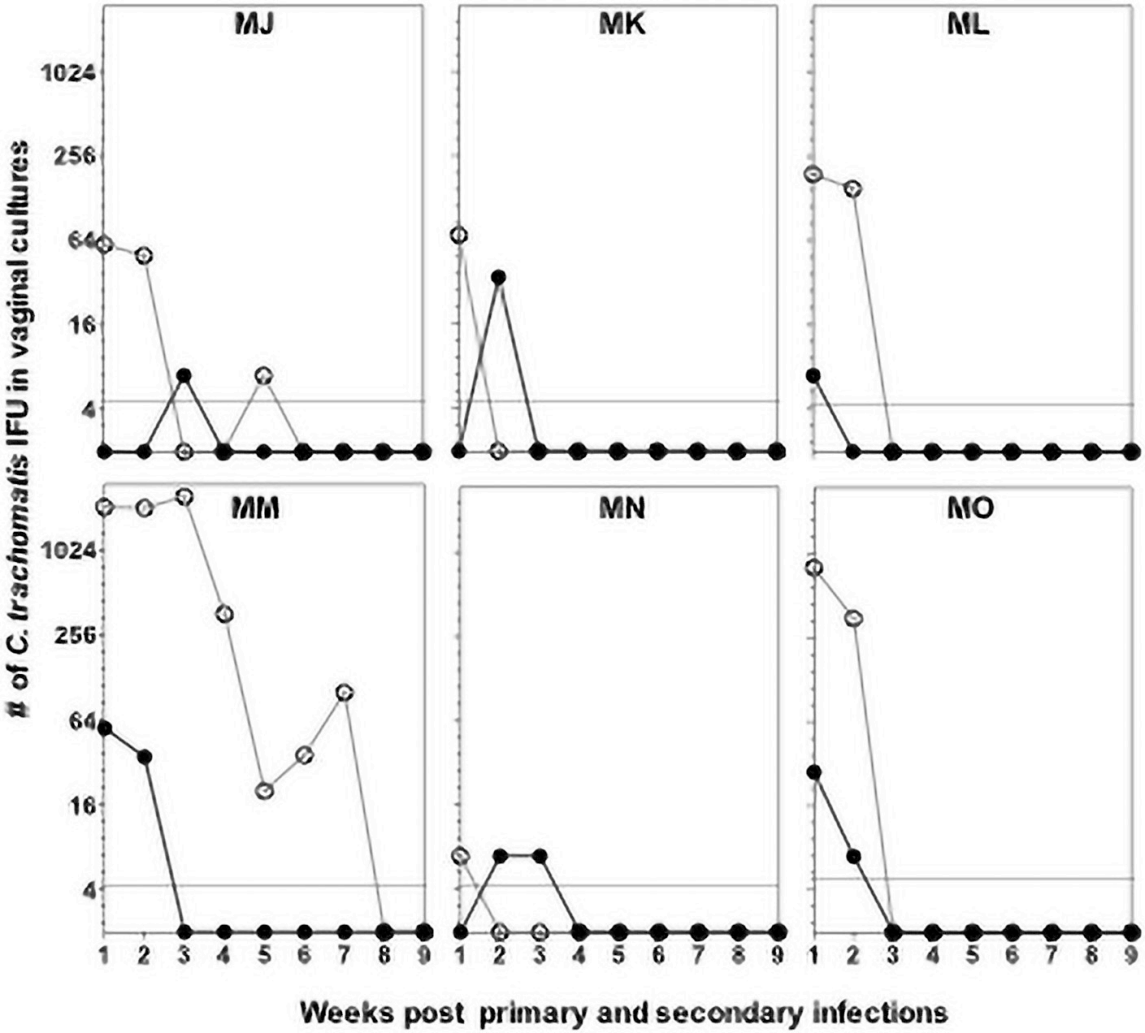
Time course of vaginal culture measurements following primary and secondary infections. The plot presents the base-2 logarithm of the number of *C*. *trachomatis* IFU in vaginal cultures from one to nine weeks after primary and secondary infections. Each unit on the y-axis represents a doubling in the raw measurement. Primary infection: empty circles and secondary infection: black circles.

Following the primary cervical *C*. *trachomatis* serovar D infection, vaginal cultures were collected at weekly intervals (Figs 1 and 2, and S1 Table). All infected monkeys had positive cultures at one-week post-infection. The number of monkeys with positive vaginal cultures subsequently declined. By week 3, 58.3% (7/12) and by week 4 only 16.7% (2/12) of the animals had positive cultures. At week 9 postinfection all the monkeys had negative vaginal cultures and remained negative until the time of the second infection. The three mock-infected monkeys had negative cultures (data not shown).

At week 14 following the primary infection, six of the animals (three initially infected with 10^7^ and three with 10^5^ IFU) were inoculated cervically with 10^5^ IFU of *C*. *trachomatis* serovar D. At week 15, 50% (3/6) of the monkeys had positive vaginal cultures and by week 16, 66.7% (4/6) were positive (Fig 1). At week 18 all cultures were negative and remained negative for an additional five weeks.

To directly compare the primary and the secondary infections only the cultures of the six monkeys infected at week 0 and again at week 14 were analyzed (Fig 2 and S1 Table). The total number of *C*. *trachomatis* IFU collected over the 8 weeks period following the primary infection (8,754) was significantly greater than the number collected after the secondary infection (189; *p*< 0.05).

### Serum antibody IgG IFA titers

To determine the serum IgG IFA titers to *C*. *trachomatis* serovar D, samples were collected before infection as a control and at week 8 following the primary and secondary infections (S1 Table). All infected monkeys had *C*. *trachomatis-*specific antibody titers at week 8 ranging from 1:100 to 1:1,600. At week 8 after the secondary infection, the IFA titers of the infected monkeys ranged from 1:50 to 1:400. No significant differences in antibody titers were observed between monkeys infected with 10^5^ or 10^7^ *C*. *trachomatis* IFU. Therefore, both groups were used for analyses. Control mock-infected monkeys were all negative (titer < 1:50; data not shown).

### Microarray preparation and sample data

The protein microarray included the expression products of 894 unique ORFs of *C*. *trachomatis* serovar D as well as positive and negative controls. Epitope tag (poly-His and HA) detection was used to verify protein expression. Of the 894 ORFs arrayed, 864 (96.4%) were positive for both the N-terminal poly-His and the C-terminal HA tags.

Microarrays were used to profile the antibody response in 72 serum samples collected from 15 rhesus monkeys. Samples were analyzed from all animals at weeks 0, 4, and 8 post-infection. Additional samples were evaluated from 6 rhesus macaques at weeks 14, 18, and 23 post-infection and 3 mock infected controls.

The dose and sample identifier information for all 72 samples are provided in S2 Table. The VSN-normalized data for the 72 samples for all 894 proteins are provided in S3 Table. Complete statistical analysis results for all 894 ORFs are shown in S4 Table.

### Serological identification of C. *trachomatis* immunodominant antigens following a primary cervical infection

**Fig 3:**
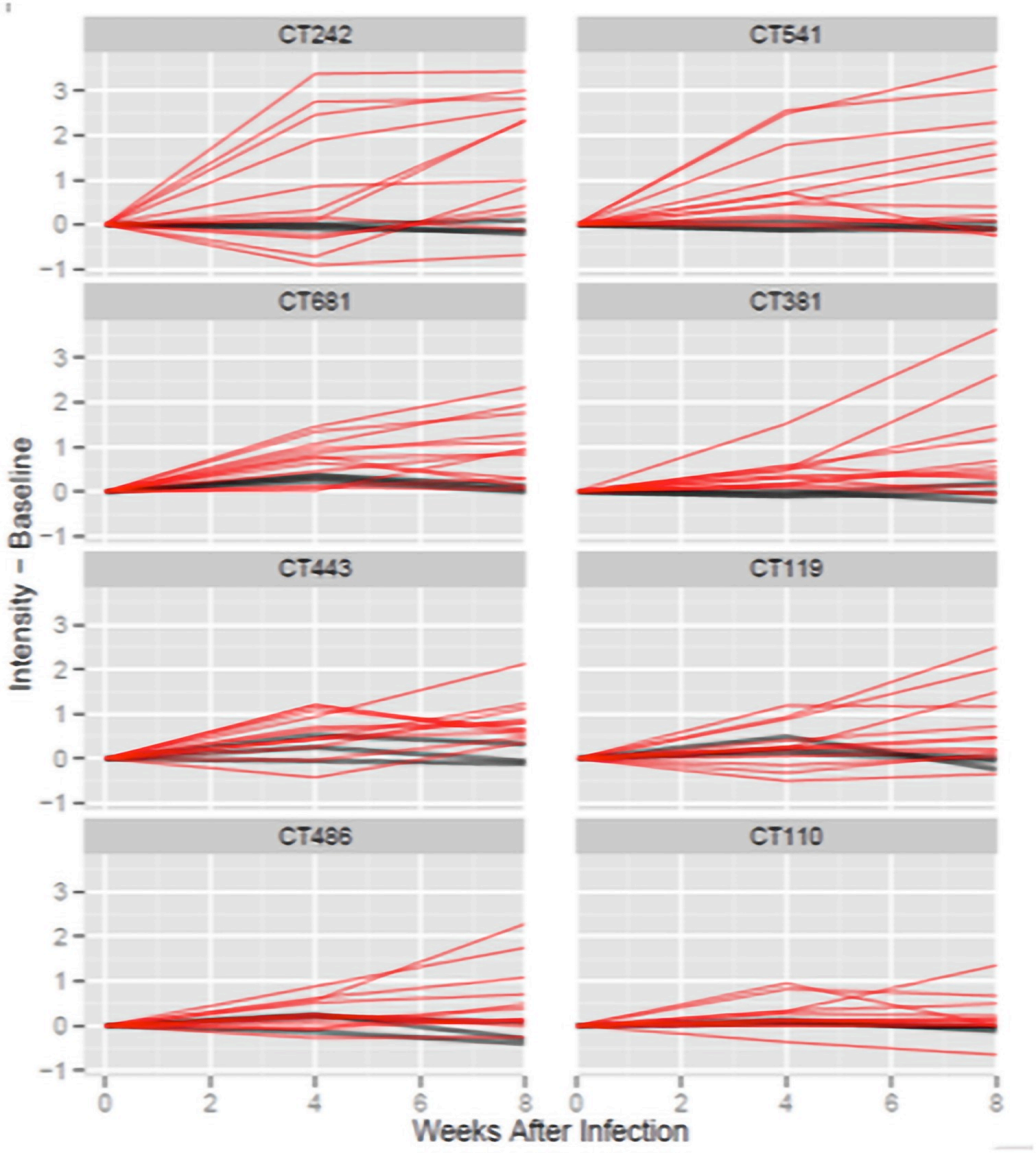
Time course of antibody responses from week 0 to week 8. Each panel presents the time course of antibody responses for all 15 monkeys for one reactive antigen. The x-axis indicates the weeks since infection and the y-axis shows the normalized intensity after subtracting the week 0 intensity for each monkey. Mock control monkeys are shown with dark gray lines (n = 3), and infected monkeys (n = 12) are shown with red lines.

The increases in normalized signal intensity from week 0 to week 8 were analyzed to identify a subset of antigens that elicited immunodominant antibody responses following a primary cervical infection (Fig 3). The criteria for selection were:

- At least 75% of the infected monkeys (9/12) showed an increase in antibody response from week 0 to week 8 that was greater than the maximum observed among the three-control mock-infected monkeys: 38 proteins met this criterion.
- The average increase in signal intensity from week 0 to week 8 was greater than 15%: 34 antigens met this criterion.

Eight antigens satisfied both criteria: CT242, CT541, CT681, CT381, CT443, CT119, CT486, and CT110. Table 1 provides basic statistics for these antigens as well as a summary of which prior studies also identified these specific antigens. S4 Table provides statistical analysis results for all 894 ORFs.

Twelve of the 15 monkeys were infected with *C*. *trachomatis* at week 0 of the study. The increases from week 0 to week 8 observed for the 12 monkeys resulted in unadjusted p-values < 0.01 for all but one of the immunodominant antigens (CT110, p = 0.08) in paired t-tests. In independent sample comparisons of the increases observed from week 0 to week 8 among the mock-infected control monkeys (n = 3) and infected monkeys (n = 12), all but one of the immunodominant antigens results in an unadjusted p-value < 0.01 (CT110, *p* = 0.04).

Among proteins not included in the filtered set of immunodominant antigens, there were 15 with paired t-test *p*-values < 0.01 and eight with independent test p-values < 0.01. Two antigens appear in both of these short lists: CT067 and CT625. Each of these proteins meets one of the filtering criteria, but not both.

### Antibodies to C. *trachomatis* immunodominant antigens increase following a secondary infection

**Fig 4:**
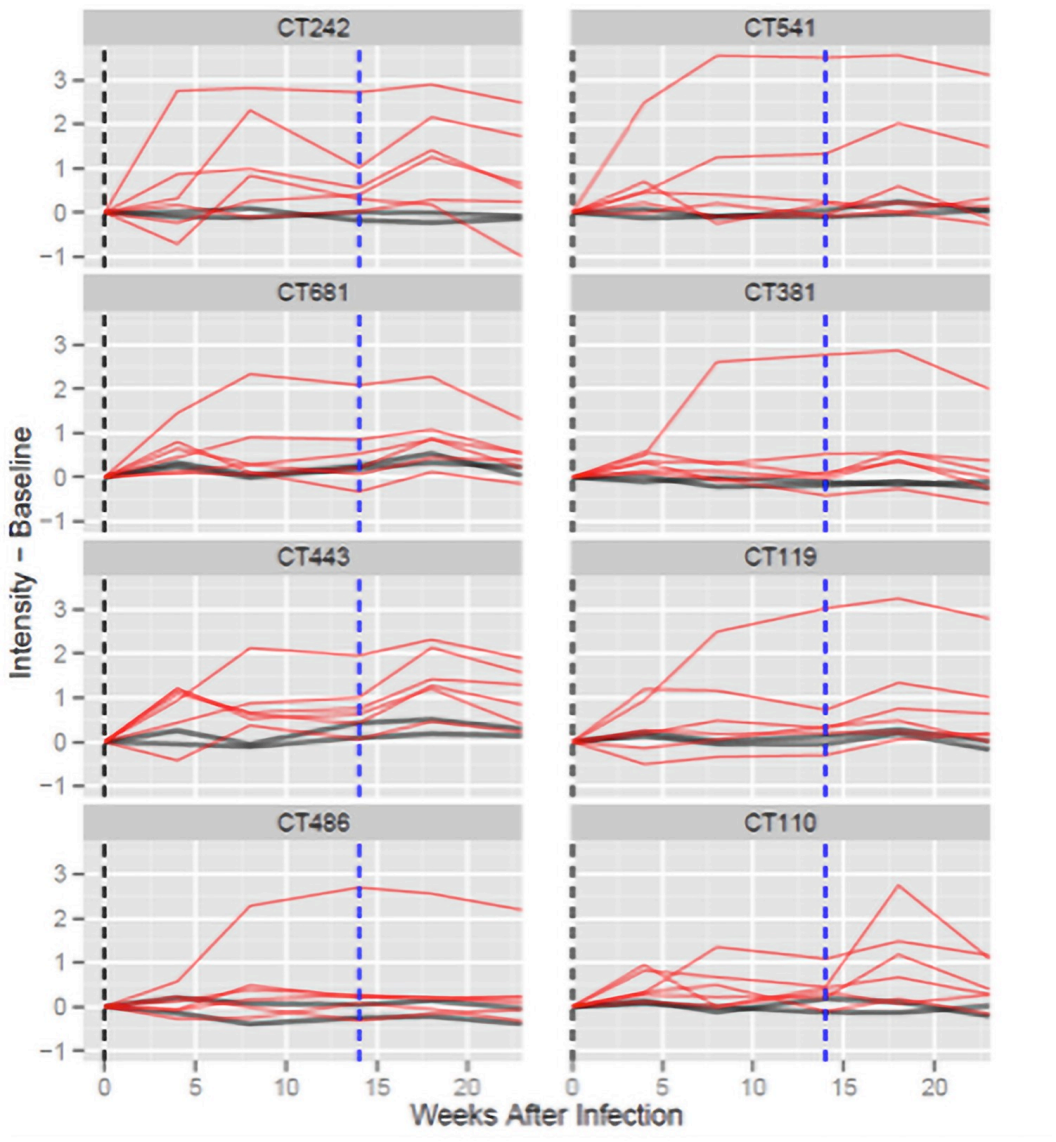
Time course of antibody responses from week 0 to week 23. Each panel presents the time course of antibody responses for one reactive antigen (six monkeys infected at weeks 0 and 14 and mock controls). The x-axis shows the weeks since infection and the y-axis presents the normalized intensity after subtracting the week 0 intensity for each monkey. Infected monkeys are shown with red lines and mock control monkeys are shown with dark gray lines. Vertical dashed lines indicate the first (black: week 0) and second (blue: week 14) infections.

Six monkeys were infected with *C*. *trachomatis* serovar D at week 0 and at week 14 (Fig 4). All but one of the immunodominant antigens had an average increase from week 14 to week 18 of at least 16% following re-infection (CT486 decreased by 3%). Among the immunodominant antigens CT443 (*p* = 0.001) and CT681 (*p* = 0.002) had increases with unadjusted *p*-value < 0.01. Nine additional antigens, not among the classified as immunodominant, also resulted in a *p*-value < 0.01. The average increases from week 14 to week 18 are shown in Table 1 for the immunodominant antigens and the increases, as well as associated *p*-values, are presented for all 894 ORFs in S4 Table.

### Time course responses following the primary and secondary infections

Fig 3 presents the responses of all 15 monkeys, including the mock-infected animals, to the eight selected immunodominant antigens at weeks 0, 4, and 8 following the primary infection. Fig 4 shows responses at weeks 0, 4, 8, 14, 18, and 23 for the six monkeys infected at weeks 0 and 14, and for the control monkeys that were mock-infected both times.

For all eight antigens, with the exception of CT110, the majority of the infected monkeys show a clear increase by week 8. The control monkeys that were mock-infected at both weeks 0 and 14 exhibited minimal change with respect to week 0 across all time points.

Overall, the most consistent increases were observed at week 8 post-infection with many similar increases from week 0 to 4 and week 4 to 8. The majority of responses to CT541 and CT681 represent good examples. The two antigens that deviate most from this trend were CT242 and CT443.

With CT242, many of the responses nearly peak at week 4 and were followed by relatively modest increases from week 4 to 8, while other responses were minimal at week 4 then show strong increases from week 4 to 8.

For CT443, most responses increased from week 0 to 4, and 4 to 8; however, three monkeys with very strong increases from week 0 to 4 dropped markedly from week 4 to 8. For other immunodominant antigens decreases from week 4 to 8 were not observed for more than two monkeys.

Following a secondary infection at week 14, most responses to the immunodominant antigens followed a consistent pattern: they increased by week 18 and then declined by week 23.

### Direct comparison of the primary and secondary infections

From week 4 after infection to the next sample the kinetics of the responses are very different after primary and secondary infections. Table 4 presents the percentages of measurements among the eight immunodominant antigens that increase, are stable, or decrease for primary and secondary infections. From the primary infection to 4 weeks later 39.6% of the signals increase, 35.4% are stable, and 25% decrease. From the secondary infection to 4 weeks later only 2.1% of signals increase, 27.1% are stable, and 70.8% decrease.

## Discussion

Here, for the first time, we have identified immunodominant antigens in sera from nonhuman primates following a primary genital infection with *C*. *trachomatis*. Sera from rhesus macaques (*Macaca mulatta*), infected once in the cervix with *C*. *trachomatis* serovar D, were used to identify immunodominant antigens that elicited a humoral immune response following a primary infection. A protein microarray, corresponding to 96.4% (864/894) of the ORFs of *C*. *trachomatis* serovar D, was used to screen the samples. Eight proteins, recognized by sera from at least 75% (9/12) of the *Chlamydia*-infected macaques, were considered to be immunodominant. This included CT110 (GroEL), CT242 (OmpH-like protein), CT443 (OmcB), CT541 (MIP) and CT681 (OmpA) that have been previously recognized as immunodominant antigens using sera from pigtailed macaques infected multiple times in the cervix, or in the oviducts, with *C*. *trachomatis* serovars D or E [35]. Proteins, not previously classified as immunodominant, using sera from nonhuman primates include CT119 (IncA), CT486 (FliY) and CT381 (ArtJ). These eight proteins may now be used to identify patients with primary *C*. *trachomatis* genital infections. If serum samples are collected on a regular basis, potentially, secondary infections could also be identified based on the antibody responses to the immunodominant antigens. These antigens can also be tested in a vaccine to determine their ability to elicit protection and may be particularly important since they are recognized following a primary *C*. *trachomatis* infection.

A primary *C*. *trachomatis* infection in humans can lead to severe bilateral pyosalpinx with tubal dilatation and long-term sequelae including infertility [43]. This is not surprising since, due to the lack of adaptive immunity, primary infections are usually more severe than reinfections, a finding observed in this and other studies [44]. In addition, young females, the population more likely to have a primary infection, frequently have cervical ectopy that makes them more susceptible to *C*. *trachomatis* [45, 46]. Similarly, young female mice are more susceptible to *C*. *muridarum* infection and infertility than older animals [47]. Likewise, most cases of PID occur within two weeks of the diagnosis of an acute *C*. *trachomatis* infection [4]. In the landmark study by Westrom et al. [7], the majority of the infertility cases 56% (79/141) occurred in women following a single PID episode. Although there are no definitive long-term longitudinal studies, the current data suggests that a significant number of cases of infertility are due to a severe primary *C*. *trachomatis* infection. Therefore, diagnosis of primary infections is critical in order to implement immediate treatment and prevent long-term sequelae.

Identification of immunodominant antigens following a primary infection with *C*. *trachomatis* in humans is not feasible. The uncertainty of the history of sexually transmitted infections makes it very difficult to diagnose a primary *C*. *trachomatis* genital infection. Equally challenging is obtaining pre-infection control serum samples. Furthermore, characterization of the immune responses to a primary *C*. *trachomatis* genital infection is difficult due, as shown here using an IFA, by the low antibody levels detected [48, 49]. In addition, the high background due to cross-reactivity with other species of *Chlamydiae* and homologous bacterial antigens, such as the heat-shock proteins, makes it challenging to evaluate serological results [6, 11, 40].

Analyses of immune responses to antigens from pathogens has significantly been facilitated by the use of high throughput screening methods. For example, to test sera from infected females Wang et al. [25] used microtiter plates coated with recombinant *C*. *trachomatis* proteins. Six of the dominant antigens they identified (CT110, CT381, CT443, CT456, CT541 and CT681) are included in our list of dominant antigens. Follmann et al. [50], using a panel of 116 recombinant *C*. *trachomatis* serovar D proteins, screened sera and peripheral blood mononuclear cells from *C*. *trachomatis* infected patients. The only two proteins recognized by both antibodies and T cells (CT110 and CT443) are part of the eight immunodominant antigens identified here. Detection of chlamydial proteins in rhesus macaques that have also been found to be immunodominant in humans validates this nonhuman primate model.

CT242 was the most immunodominant antigen detected since it was recognized by sera from all infected monkeys and elicited the highest antibody levels. This protein is likely a member of the OmpH (Skp) family reported in other bacteria to have a chaperonin activity critical for the biosynthesis of the outer membrane [51]. CT242 was first found to elicit strong antibody responses in male cynomolgus monkeys (*Macaca fascicularis*) following a urethral infection with *C*. *trachomatis* serovar L2 (LGV-434) [52]. In addition, CT242 was shown by Coler et al. [53] to stimulate peripheral blood mononuclear cells from *C*. *trachomatis* infected patients. CT242 and its ortholog in *Chlamydia pneumoniae* (CPn0301), are located in the outer membrane of *Chlamydiae* [52, 54]. Interestingly, the CPn0301 protein has been shown to elicit protection in hamsters against an intraperitoneal challenge with *C*. *pneumoniae* [55].

A strength of this study is that all monkeys were only infected with *C*. *trachomatis* serovar D, the same isolate used to generate the microarray. Most published studies were performed using antigens from *C*. *trachomatis* serovar D since the whole genome sequence is known [56]. There is over 98% DNA homology between the 15 *C*. *trachomatis* serovars, however SNPs or other mutations may be present. This may bias the results when testing serum samples from patients that may have been infected with serovars other than D or with multiple serovars.

This approach may have some limitations. For example, the expressed proteins do not include post-translational modifications and therefore, antibodies recognized by those structures may not be identified in this assay. However, as previously shown in a vaccinia virus array prepared by this method, all known glycosylated proteins were recognized by sera from immunized humans and animals. [57]. Conformational epitopes and those formed by disulfide bonds may not be present in this microarray [58–60]. Monoclonal antibodies to linear epitopes, but not to conformational epitopes, recognize MOMP (CT681) in this microarray (unpublished data). Here we detected MOMP indicating that antibodies to linear peptides were generated [61, 62].

In order to directly compare the effects on serological responses in a primary and a secondary infection here we restricted the analyses to the six monkeys that were infected and then reinfected. We do not know to what degree the observations made in this controlled study with monkeys will translate to human infections. However, the data from this study offers enough evidence to construct ELISA with the 8-antigens highlighted. In some clinical studies, pre-infection samples are obtained and serial samples are taken. In the context of serial sampling the kinetics of antibody responses to the protein antigens highlighted here may be used to differentiate primary from secondary chlamydial infections. In a primary infection the antibody levels are still increasing 8 weeks after infection whereas in the secondary infection they peak 4 weeks after infection and then decline. Furthermore, in the primary infection the responses observed 4 weeks after infection are weaker than those observed 4 weeks after reinfection.

Humans frequently have STI simultaneously with several pathogens that, in addition to *C*. *trachomatis*, can include herpes simplex viruses, human papillomaviruses, *Mycoplasma genitalium*, *Neisseria gonorrhoeae* and *Treponema pallidum* [1, 2]. Also, the normal vaginal and cervical flora varies from individual to individual. How the different types of genital flora will affect the antibody responses of an individual to a particular pathogen is unknown. Considering the different immunogenetic backgrounds of each patient and the multiple variables that the different combinations of various pathogens can create, gaining an understanding on how the antibody responses under the different circumstances could vary will be very difficult to assess.

In conclusion, using sera from rhesus macaques collected following a primary cervical *C*. *trachomatis* infection, we have identified antigens that can be used to screen patients to determine if this is the first time they were infected with this pathogen. In animal models, these immunodominant antigens can also be tested for their ability to protect against a chlamydial infection before implementation of a vaccine in humans.

## Supporting information

S1 Table Vaginal cultures and IFA titers in serum. Total number of *C*. *trachomatis* IFU collected from the cervix at 8-weeks following the primary and the secondary infections. Serum IFA IgG titers against *C*. *trachomatis* serovar D before infection and at week-8 following the primary and the secondary infections.
(DOCX)

S2 Table Dose and sample information. Primary (week 0) and secondary (week 14) infection doses and sample identifiers for all 15 monkeys.
(XLS)

S3 Table VSN normalized data. (CSV)

S4 Table Complete statistics. Average difference and associated t-test p-value for several comparisons of time points from the same monkeys (paired comparisons) and control versus infected (independent comparisons) for all 894 ORFs.
(CSV)
