# Supplementary material for: A primary Chlamydia trachomatis genital infection of rhesus macaques identifies new immunodominant B-cell antigens
Source: PLoS One. 2021 Apr 22;16(4):e0250317. doi: 10.1371/journal.pone.0250317 (PMC8061917; doi:10.1371/journal.pone.0250317)
Supplement: S1 Table — Total number of C. trachomatis IFU collected from the cervix at 8-weeks following the primary and the secondary infections. Serum IFA IgG titers against C. trachomatis serovar D before infection and at week-8 following the primary and the secondary infections. (DOCX) [file pone.0250317.s001.docx]

**S1 Table. Serum IgG IFA titers and number of *C. trachomatis* IFU recovered from vaginal cultures following a primary and a secondary infection.**

| **Primary infection** | | | | | |  | **Secondary infection** | | | | |
| --- | --- | --- | --- | --- | --- | --- | --- | --- | --- | --- | --- |
| Monkey  ID | IFA titers before  infection | Infection  dose  wk0 | #Wks to  clear infection | Total #IFU recovered from 8 wks  of cultures | IFA titers  8 wk p.i. |  | IFA titers  before  infection | Infection  dose  wks14 | #Wks to  clear infection | Total #IFU recovered from 8 wks  of cultures | IFA titer  22 wks p.i. |
| MD | <50 | 10^7^ | 4 | 140 | 200 |  | Euth^a^ |  |  |  |  |
| ME | <50 | 10^7^ | 5 | 380 | 400 |  | Euth^a^ |  |  |  |  |
| MF | <50 | 10^7^ | 6 | 67 | 1,600 |  | Euth^a^ |  |  |  |  |
| MG | <50 | 10^5^ | 4 | 270 | 400 |  | Euth^a^ |  |  |  |  |
| MH | <50 | 10^5^ | 9 | 664 | 200 |  | Euth^a^ |  |  |  |  |
| MI | <50 | 10^5^ | 5 | 1,897 | 400 |  | Euth^a^ |  |  |  |  |
| MJ | <50 | 10^7^ | 6 | 117 | 200 |  | 200 | 10^5^ | 4 | 7 | 200 |
| MK | <50 | 10^7^ | 2 | 70 | 200 |  | 50 | 10^5^ | 3 | 35 | 50 |
| ML | <50 | 10^7^ | 3 | 340 | 100 |  | 100 | 10^5^ | 2 | 7 | 200 |
| MM | <50 | 10^5^ | 8 | 7,068 | 400 |  | 200 | 10^5^ | 3 | 91 | 100 |
| MN | <50 | 10^5^ | 2 | 7 | 100 |  | 50 | 10^5^ | 4 | 14 | 400 |
| MO | <50 | 10^5^ | 4 | 1,152 | 400 |  | 200 | 10^5^ | 3 | 35 | 400 |

^a^ Euthanized.
